# Multiple vascular anomalies and refractory pericardial effusion in a young patient with Cantu syndrome: a case report and review of the literature

**DOI:** 10.1186/s12887-023-04446-8

**Published:** 2023-12-19

**Authors:** Falastine Daas, Punita Gupta, Fuad Kiblawi

**Affiliations:** 1 Department of Pediatrics, St. Joseph’s University Medical Center, 703 Main Street, Paterson, NJ 07503 USA; 2 Department of Pediatrics Division of Genetics, St. Joseph’s University Medical Center, 703 Main Street, Paterson, NJ 07503 USA; 3 Department of Pediatrics Division of Cardiology, St. Joseph’s University Medical Center, 703 Main Street, Paterson, NJ 07503 USA

**Keywords:** Cantu syndrome, Pericardial effusion, *ABCC9* gene, *KCNJ8* gene, Lymphedema, Hypertrichosis, Left ventricular hypertrophy, Cardiomegaly, Aortic root dilation

## Abstract

**Background:**

Cantu syndrome is a rare and complex multisystem disorder characterized by hypertrichosis, facial dysmorphism, osteochondroplasia and cardiac abnormalities. With only 150 cases reported worldwide, Cantu syndrome is now gaining wider recognition due to molecular testing and a growing body of literature that further characterizes the syndrome and some of its most important features. Cardiovascular pathology previously described in the literature include cardiomegaly, pericardial effusion, vascular dilation and tortuosity, and other congenital heart defects. However, cardiovascular involvement is highly variable amongst individuals with Cantu syndrome. In some instances, it can be extensive and severe requiring surgical management and long term follow up.

**Case presentation:**

Herein we report a case of a fourteen-year-old female who presented with worsening pericardial effusion of unknown etiology, and echocardiographic findings of concentric left ventricular hypertrophy, a mildly dilated aortic root and ascending aorta. Her medical history was notable for hemoptysis and an episode of pulmonary hemorrhage secondary to multiple aortopulmonary collaterals that were subsequently embolized in early childhood. She was initially managed with Ibuprofen and Colchicine but continued to worsen, and ultimately required a pericardial window for the management of refractory pericardial effusion. Imaging studies obtained on subsequent visits revealed multiple dilated and tortuous blood vessels in the head, neck, chest, and pelvis. A cardiomyopathy molecular studies panel was sent, and a pathogenic variant was identified in the *ABCC9* gene, confirming the molecular diagnosis of autosomal dominant Cantu syndrome.

**Conclusions:**

Vascular anomalies and significant cardiac involvement are often present in Cantu syndrome, however there are currently no established screening recommendations or surveillance protocols in place. The triad of hypertrichosis, facial dysmorphism, and unexplained cardiovascular involvement in any patient should raise suspicion for Cantu syndrome and warrant further investigation. Initial cardiac evaluation and follow up should be indicated in any patient with a clinical and/or molecular diagnosis of Cantu syndrome. Furthermore, whole body imaging should be utilized to evaluate the extent of vascular involvement and dictate long term monitoring and care.

## Background

Cantu syndrome (CS) is a rare genetic disorder originally described by Cantu et al. in 1982 with only about 150 cases described worldwide to date [1]. It is an autosomal dominant disorder characterized by hypertrichosis, macrocephaly and coarse facial features with involvement of the skeletal, cardiovascular, lymphatic and pulmonary systems. Cantu syndrome arises from mutations in either the *ABCC9* or the *KCNJ8* gene resulting in the dysregulation of the ATP-sensitive potassium channel [2]. Some individuals with a clinical diagnosis of Cantu syndrome have not had a pathogenic variant identified in either gene, suggesting the existence of another as-yet unidentified causative gene.

Cantu syndrome is now gaining wider recognition with about 150 known cases worldwide and molecular identification of the pathogenic mutation in 107 patients [1]. There is multi-system involvement and a wide range of disease phenotypes and severity that makes a timely diagnosis challenging. Cardiovascular involvement is commonly seen in patients with Cantu syndrome and can be associated with significant cardiac and systemic vascular anomalies. Herein we present a case of a fourteen-year-old female with significant cardiovascular involvement namely refractory pericardial effusion, multiple dilated blood vessels and hemoptysis secondary to the presence of aortopulmonary collaterals. Her diagnosis remained elusive for ten years until a cardiomyopathy molecular genetic panel was obtained, which identified a pathogenic variant in the *ABCC9* gene, confirming the diagnosis of Cantu syndrome.

## Case presentation

The patient is the first child of non-consanguineous parents, born full term via induced vaginal delivery following an uncomplicated pregnancy and benign perinatal course. Her early developmental milestones were somewhat delayed. She spoke her first words around one year of age, was combining 2 words at 18 months of age, and walked independently at 16 months of age. She had mild learning difficulties and was receiving speech therapy in school. Her past medical history was significant for recurrent sinusitis, recurrent otitis media, tonsillitis and one episode of pneumonia. Her physical examination was remarkable for multiple dysmorphic features, including coarse facial features, with a prominent nasal bridge and anteverted nares. She was noted to have hypertrichosis of the forehead, face, arms, legs, and lower back. Her musculoskeletal examination was significant for a narrow thorax, joint laxity in her fingers and elbows, short broad feet with short toes and very tight heel cords. She was evaluated for Hurler-Scheie-like Syndrome at three years of age due to suggestive facial features, however metabolic and molecular testing were negative.

At five years of age, she developed subacute hemoptysis over the course of several weeks and one episode of large hemoptysis necessitating hospital admission and evaluation. She underwent bronchoscopy that was complicated by massive pulmonary hemorrhage and required intubation and subsequent transfer to a large tertiary center. CT angiography was done which revealed two abnormal blood vessels as the likely etiology of her symptoms. She eventually underwent cardiac catheterization and was found to have several aortopulmonary collaterals that required transcather occlusion. Hemodynamic assessment yielded elevated right-sided pressures consistent with pulmonary hypertension. She was admitted for a total of three weeks and continued to follow up with the pulmonary hypertension clinic and cardiologist for the following three years but unfortunately had been lost to follow-up thereafter.

**Fig. 1:**
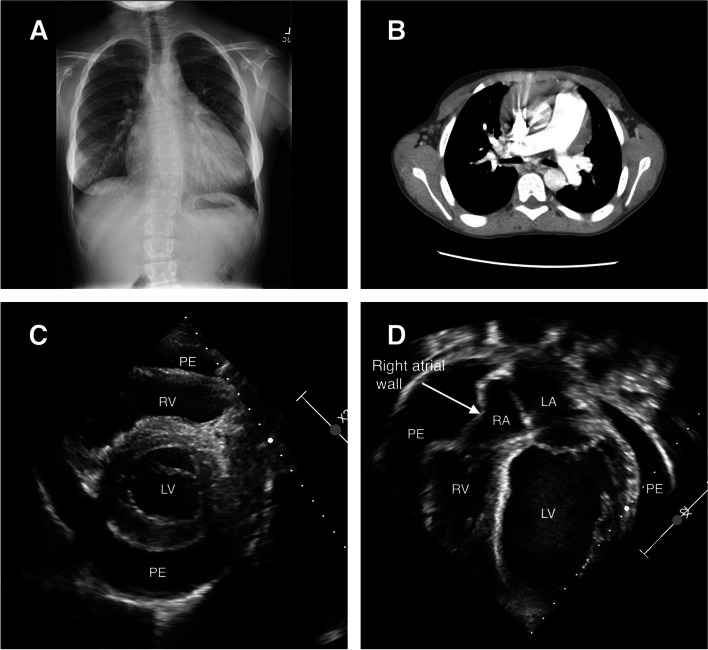
**A** Chest xray revealing an enlarged cardiomediastinal silhouette, levoscoliosis of the thoracic spine and dextroscoliosis of the lumber spine. **B** CT pulmonary angiogram showing a dilated pulmonary trunk measuring at least 3.5 c.m with prominent peripheral pulmonary arteries. **C** Transthoracic echocardiogram in the parasternal short axis view revealing pericardial effusion. **D** Transthoracic echocardiogram in the apical view depicting pericardial effusion and right atrial wall compression. LA: Left Atrium, LV: Left Ventricle, PE: Pericardial Effusion, RA: Right Atrium, RV: Right Ventricle

She remained in relatively good health with no recurrence of hemoptysis and presented to our institution for cardiac evaluation at 11 years of age. An echocardiogram was obtained at that visit which revealed a patent foramen ovale with left to right flow, mild concentric left ventricular hypertrophy, a mildly dilated aortic root and ascending aorta in addition to a small pericardial effusion. She was evaluated by pediatric pulmonology in the interim and a chest x-ray and CT pulmonary angiography of the chest was obtained which revealed cardiomegaly, scoliosis and a dilated pulmonary trunk and peripheral pulmonary arteries (Fig. 1A, B). Repeat echocardiogram done six months later demonstrated left ventricular hypertrophy and a small to moderate circumferential pericardial effusion which had increased compared to the prior study. Although asymptomatic, she was referred to pediatric rheumatology for further evaluation given the uncertain etiology of the pericardial effusion. Subsequent rheumatologic evaluation was negative, however she continued to have progressively worsening pericardial effusion on serial echocardiograms despite management with Ibuprofen, Colchicine and Prednisone. Repeat imaging demonstrated worsening pericardial effusion and intermittent right atrial collapse (Fig. 1C, D). Due to these findings, she was ultimately admitted for a video-assisted thoracoscopic surgery (VATS), pericardial window and chest tube placement. Pathologic evaluation of the pericardium revealed benign fibroconnective tissue with focal congestion. Cytology of pericardial fluid did not reveal any malignant cells.

**Fig. 2:**
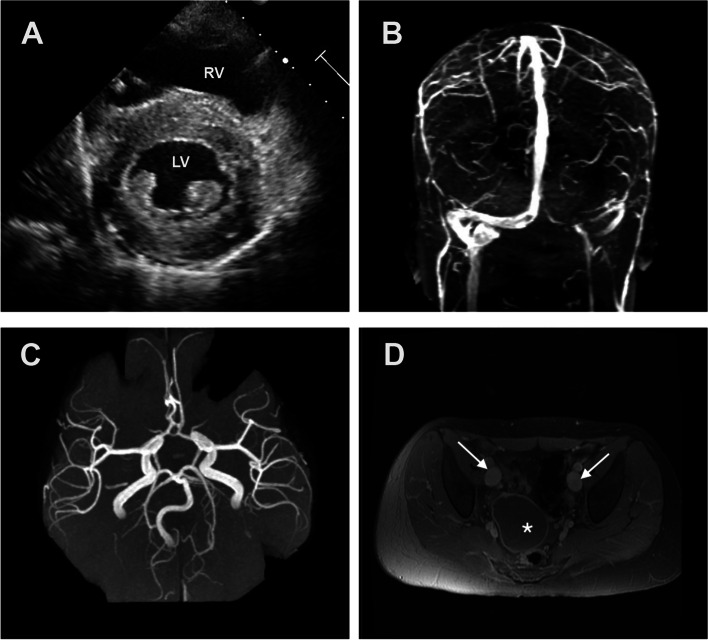
**A** Transthoracic enchocardiogram in the parasternal short axis view depicting left ventricular hypertrophy. **B** and **C** MRV and MRA revealing dilated and slightly tortuous dural veins and arteries of the circle of Willis. **D** MRI of the pelvis in the axial plane post contrast depicting dilated bilateral iliac veins (arrows) and a right adnexal cystic mass with rim enhancement (asterisk). LV: Left Ventricle, RV: Right Ventricle

The patient had a benign post-operative course and was seen for follow-up cardiac evaluation after discharge. Repeat echocardiogram at that point revealed mild left ventricular hypertrophy (Fig. 2A), normal left ventricular systolic function and complete resolution of the previously noted pericardial effusion. However, her physical examination at that time revealed mild-moderate bilateral lymphedema which was not previously noted on prior examinations. Given the constellation of cardiovascular findings namely the left ventricular hypertrophy of unknown etiology, large pericardial effusion, history of aorto-pulmonary collaterals and new onset lymphedema in a patient with non-specific dysmorphic features, a cardiomyopathy molecular studies panel was sent, and a pathogenic variant was identified in the *ABCC9* gene, confirming the molecular diagnosis of autosomal dominant Cantu syndrome. In light of the diagnosis of Cantu syndrome the patient was referred for further genetic evaluation and counseling. On her most recent follow-up, our patient was noted to have persistent headaches and underwent brain MRI with vascular imaging which revealed dilated and slightly tortuous dural veins and arteries of the circle of Willis (Fig. 2B, C). MRI of the neck, chest, and pelvis revealed dilated subclavian and axillary veins and arteries bilaterally, dilated main and peripheral pulmonary arteries, and dilated iliac veins bilaterally (Fig. 2D). She was also incidentally noted to have a right adnexal cystic mass of uncertain clinical significance (Fig. 2D). Given these findings, she was referred for evaluation by pediatric neurology and neurosurgery. Moving forward, our patient will likely need follow up with a multidisciplinary team in order to address her collective symptoms and findings.

## Discussion and conclusions

### Genetics of Cantu syndrome

Previous studies and initial reports indicate that a pathogenic variant in either *ABCC9* or *KCNJ8* gene is the primary cause of Cantu syndrome [1]. Although initial studies suggested an autosomal recessive pattern, segregation analysis by Robertson et al. revealed autosomal dominant inheritance with many cases occurring due to de novo mutations [3, 4]. This is evident in our case report, with our patient’s parents testing negative for the pathogenic variant of the *ABCC9* gene confirming the suspicion that her clinical condition results from a de novo mutation. To date, there are only four known cases attributed to heterozygous pathogenic variants in the *KCNJ8* gene [5–8]. The remaining cases with identifiable mutations carry heterozygous pathogenic variants of the *ABCC9* gene. Some individuals with a clinical diagnosis of Cantu syndrome have not had a pathogenic variant identified in either gene, suggesting the existence of another as-yet unidentified causative gene.

One individual identified by the Cantu syndrome International Registry (ICSR) displayed somatic and germline mosaicism with relatively mild features and no evidence of systemic involvement. He was only later identified through molecular testing after the diagnosis of a severely affected daughter [1]. This carries the implication that perhaps family members with milder features may not be identified due to the absence of defining features of Cantu syndrome. Furthermore, this suggests that Cantu syndrome patients could fall within a spectrum of disease severity as indicated by variable expression of symptoms even among affected members of the same family [9].

Knockout mouse models of Cantu syndrome have demonstrated more severe clinical features in the *KCNJ8* variants compared to the *ABCC9*-related variants [10]. The low incidence of *KCNJ8* variants coupled with the severe clinical features in such patients makes it possible that *KCNJ8* mutations may be detrimental [1]. Our case presentation supports the notion that affected individuals with *ABCC9* mutations could certainly develop progressive and severe symptoms over time. To date, more than 30 different missense mutations have been identified in Cantu syndrome [11]. At this time, we cannot predict the clinical course or disease severity solely based on knowledge of the genetic mutations present. Moving forward, a larger cohort of patients is required to draw conclusions regarding genotype–phenotype relationships.

### Cardiovascular involvement: important features and pathophysiology

Cardiovascular involvement is frequently reported in individuals with Cantu syndrome and can include cardiomegaly, pulmonary hypertension, pericardial effusion, dilated and tortuous blood vessels and congenital heart defects [12]. Cardiomegaly in Cantu syndrome is characterized by ventricular enlargement with preserved or increased systolic function (high output state) in which muscle function and histology are typically normal [9, 13]. Cantu syndrome is caused by gain of function mutation of the *ABCC9* or *KCNJ8* genes which encode the SUR2 (sulfonylurea receptor) and pore-forming Kir6.1 subunits of the ATP-sensitive potassium channels respectively, which translates to K-ATP channel overactivity [7, 15]. Kir6.1 and SUR2 are co-expressed in the heart, skeletal and smooth muscle cells and consequently, their dysfunction can lead to multi-system manifestations not limited to the cardiovascular system. Involvement of vascular smooth muscles can result in chronically dilated vessels with subsequent hypotension and reduction in vascular tone [10, 12, 14]. It is postulated that the chronic reduction in systemic vascular resistance results in elevated Renin-Angiotensin signaling which in turn increases blood volume and promotes cardiac enlargement [15]. High output, hypertrophic cardiac remodeling thus arises secondary to systemic vasodilation as a compensatory mechanism to normalize tissue perfusion [14]. This could potentially result in decreased cardiac reserve and exercise tolerance in affected individuals. However, further research is needed to fully understand the long-term consequences of cardiac remodeling in Cantu syndrome patients.

Progressive pericardial effusion in patients with Cantu syndrome can lead to fatigue, dyspnea and reduced exercise tolerance and may therefore necessitate further intervention. In some extreme cases, patients have required intervention in the form of repeated pericardiocentesis, pericardial stripping or pericardial window [9, 16]. Our patient initially presented with evidence of a small pericardial effusion on echocardiogram, which became progressively worse despite medical management, resulting in intermittent right atrial collapse. Generalized edema is also often noted after birth and lymphedema of the lower extremities may develop later in life. This is thought to arise as a result of dilated lymphatic vessels in the legs along with delayed lymphatic drainage [2, 17]. There is a possible link between pericardial effusion and lymphedema that remains poorly understood. Increased blood volume or perhaps reduced cardiac lymphatic drainage have been implicated as possible mechanisms for persistent pericardial effusion in Cantu syndrome [1]. Although there have been several cases describing Cantu syndrome as a rare etiology for pericardial effusion, there are only a handful of cases in which invasive management has been required. Our case serves as a reminder that regular monitoring for pericardial effusion and cardiomyopathy in Cantu syndrome is essential as cardiovascular involvement can be severe and may not be amenable to medical therapy alone.

There is a growing body of literature that describes vascular abnormalities both within and outside the chest in patients with Cantu syndrome. Several case reports and case series have described such anomalies including aortic root dilation, aortic aneurysms, aortopulmonary collaterals, arterial-venous malformations (AVMs) and dilated and tortuous blood vessels in the head, neck, chest and abdomen [5, 12, 19, 20]. In some instances, patients have presented with persistent patent ductus arteriosus (PDA) that required surgical intervention for closure [5, 12]. Parrott et al. described longitudinal vascular findings in three individuals with Cantu syndrome and stressed the importance of regular monitoring and surveillance due to the risk of progressive aortic dilation and aortic dissection [12]. Smaller vasculature such as retinal vessels can also be involved and are also found to be dilated and tortuous in some patients with Cantu syndrome. Kisilevsky et al. described a case of 45-year-old female who was found to have dilated and tortuous retinal vasculature on routine ophthalmologic evaluation, who was only later diagnosed with Cantu syndrome [20]. Our patient was found to have significant hemoptysis as a result of multiple major aortopulmonary collaterals and required transcatheter occlusion at a fairly young age. Major Aortopulmonary collateral arteries (MAPCAs) are congenital vessels that arise from the aorta or its first order branches and are distally connected to the pulmonary vasculature thereby providing pulmonary blood flow in early embryonic life [18]. These arteries typically regress as the normal pulmonary arteries develop. It is postulated that the persistence of a PDA and MAPCAs after fetal life may arise as a result of maintained vessel dilation following birth in the setting of an abnormal/hyperactive K-ATP channelopathy [2].

Leon Guerrero et al. described neurologic and neuroimaging findings in 10 patients with Cantu syndrome who underwent vascular imaging and brain MRI studies [19]. These findings include diffusely dilated and tortuous cerebral blood vessels accompanied by white matter changes seen on brain MRI. The implications of such findings relating to neurodevelopmental outcomes is difficult to elucidate in the absence of further longitudinal studies. However, there is sufficient information in the literature to suggest the importance of early screening for vascular involvement in Cantu syndrome, particularly when the risk of disease progression remains uncertain [1].

There are currently no known targeted therapies that to address the clinical manifestations of Cantu syndrome. Some studies have investigated the potential role for KATP antagonists such as glibenclamide and tolbutamide in mitigating or reversing the cardiovascular abnormalities seen in Cantu patients. McClenaghan et al. studied the potential role of glibenclamide in reversing cardiovascular remodeling in genetically modified Cantu mice [21]. The study revealed a dose dependent reversal in cardiac hypertrophy and an elevation in mean arterial pressure (MAP). However, the study also demonstrated that vascular structural abnormalities and phenotypes secondary to the KCNJ8 mutation may be refractory to KATP inhibition. With a very limited body of literature and a few studies done in animal models, it is currently difficult to speculate on the potential clinical role of KATP channel inhibitors. There is certainly a need for clinical trials and more selective and targeted therapeutics that address specific components of the disease.

Our case in conjunction with multiple prior case reports highlights the potential for extensive cardiovascular involvement in patients with Cantu syndrome. Our patient demonstrated somewhat of a severe clinical course requiring multiple interventions over a period of ten years before a final diagnosis was made. She demonstrated a wide constellation of cardiovascular abnormalities including cardiomegaly, concentric LVH, pericardial effusion, lymphedema and dilated and tortuous blood vessels in the head, neck, chest, and pelvis. Additionally, her medical history was notable for hemoptysis secondary to MAPCAs requiring transcather occlusion, which points to the potential for persistent fetal circulation in such patients.

Despite the availability of compelling evidence that the cardiovascular system is almost always involved in Cantu syndrome, additional information is needed to guide evidence-based screening recommendations and long-term follow-up. Cardiac surveillance is imperative in such patients, and clinicians should remain cognizant of the potential for unique vascular abnormalities and lymphatic system involvement. Initial cardiac evaluation should be indicated to screen for cardiac abnormalities such as persistent PDA, MAPCAs, pulmonary hypertension, cardiomyopathy and pericardial effusion. Management and follow-up would be determined by the findings of the initial assessment. An echocardiogram should be considered annually or more frequently as dictated by the findings on baseline cardiac evaluation. As such, we recommend the following initial evaluation, follow up, and referrals summarized in Table 1 in individuals with a clinical or molecular diagnosis of Cantu syndrome. Although obligatory features in Cantu Syndrome are relatively unknown, a constellation of findings including hypertrichosis, coarse facial features and unexplained cardiovascular involvement in any patient should raise suspicion for Cantu syndrome and warrant further investigation.

**Table 1:** Recommended initial evaluation, follow up, and referrals in individuals with Cantu Syndrome

System/concern	Evaluation	Follow up	Referral
Cardiovascular	Baseline echocardiogram to evaluate for cardiomyopathy, PDA, MAPCAs, pericardial effusion, dilated aortic root, pulmonary hypertension and structural heart defects	• Annual surveillance echocardiogram • More frequent echocardiograms at the discretion of the cardiologist depending on the findings of the initial cardiac evaluation	Referral to cardiologist at the time of diagnosis
	Consider whole body imaging including MRI/MRA of the chest, abdomen and pelvis to evaluate for dilated vasculature	• Surveillance MRI/MRA every 3–5 years if initial imaging is normal • More frequent MRI/MRA at the discretion of the specialist if imaging reveals dilated and/or tortuous vasculature	Consider referral to vascular surgery as dictated by findings on imaging
Neurovascular	Consider neuroimaging including brain MRI with MRA/MRV in the setting of headaches and/or neurologic findings to evaluate for dilated/tortuous cerebral vasculature or other abnormalities	Frequency of brain MRI with MRA/MRV should be dictated by the specialist if initial imaging is abnormal or neurologic findings emerge	Referral to neurologist if neuroimaging is abnormal or neurologic manifestations including headaches are present
Microvascular abnormalities	Evaluation by an ophthalmologist to screen for dilated/tortuous retinal vessels and other abnormalities	Annual eye exam	Referral to ophthalmologist at the time of diagnosis
Lymphatic	Physical exam with particular attention to lymphedema	Annual physical exam	Consider referral to lymphedema clinic

*PDA* Persistent ductus arteriosus, *MAPCAs* Major aortopulmonary collateral arteries, *MRI* Magnetic resonance imaging, *MRA* Magnetic resonance angiography, *MRV* Magnetic resonance venography

## Conclusion

The case highlights the diagnostic challenge in evaluating patients with Cantu syndrome due to the complex and variable clinical manifestations that may develop over time. The cardiovascular system is commonly affected in patients with Cantu syndrome and unfortunately screening and surveillance protocols are still lacking. Our case report contributes to the growing body of information regarding cardiovascular involvement in Cantu syndrome and provides further insight into the possible long-term sequelae of this disease. The availability of molecular testing coupled with increased awareness of the syndrome and its features could lead to easier identification of affected individuals. Well-defined diagnostic criteria could improve recognition of the most common features of Cantu syndrome and reduce unnecessary delays in diagnosis.

## Data Availability

Data sharing does not apply to this article as no datasets were generated or analyzed during the current study.

## Abbreviations

AVM: Arterial venous malformation
CS: Cantu syndrome
LA: Left atrium
LV: Left ventricle
LVH: Left ventricular hypertrophy
MAP: Mean arterial pressure
MAPCA: Major aortopulmonary collateral-arteries
MRA: Magnetic resonance angiography
MRI: Magnetic resonance imaging
MRV: Magnetic resonance venography
PDA: Persistent ductus arteriosus
RA: Right atrium
RV: Right ventricle
VATS: Video-assisted thoracoscopic surgery
